# Inhibition of Serine Proteases as a Novel Therapeutic Strategy for Abdominal Pain in IBS

**DOI:** 10.3389/fphys.2022.880422

**Published:** 2022-05-19

**Authors:** Lisse Decraecker, Guy Boeckxstaens, Alexandre Denadai-Souza

**Affiliations:** Laboratory of Intestinal Neuro-immune Interaction, Translational Research Center for Gastrointestinal Disorders, Department of Chronic Diseases, Metabolism and Ageing, KU Leuven, Leuven, Belgium

**Keywords:** irritable bowel syndrome, visceral hypersensitivity, proteases, protease-activated receptors, protease inhibitors

## Abstract

Serine proteases are heavily present in the gastrointestinal tract where they are essential in numerous physiological processes. An imbalance in the proteolytic activity is a central mechanism underlying abdominal pain in irritable bowel syndrome (IBS). Therefore, protease inhibitors are emerging as a promising therapeutic tool to manage abdominal pain in this functional gastrointestinal disorder. With this review, we provide an up-to-date overview of the implications of serine proteases in the development of abdominal pain in IBS, along with a critical assessment of the current developments and prospects of protease inhibitors as a therapeutic tool. In particular, we highlight the current knowledge gap concerning the identity of dysregulated serine proteases that are released by the rectal mucosa of IBS patients. Finally, we suggest a workflow with state-of-the-art techniques that will help address the knowledge gap, guiding future research towards the development of more effective and selective protease inhibitors to manage abdominal pain in IBS.

## Introduction

Irritable bowel syndrome (IBS) is a highly prevalent disorder of the gut-brain axis affecting 4% of the world population (Sperber et al., 2021). Also according to the Rome IV criteria, IBS is diagnosed when a patient has recurrent abdominal pain that is associated with a change in stool frequency and/or form (Drossman, 2016). Also bloating, gas and cramping are common symptoms, but their presence is not necessary for diagnosis. IBS prevalence is higher in women than in men; female-to-male odds ratio of 1.7 (1.5–1.9), while geographical location does not seem to have an impact (Sperber et al., 2021). Effective therapy to manage the debilitating symptoms is however still lacking and the costs associated with diagnosis and work absenteeism place a heavy burden on both the patient and the health care systems (Drossman et al., 2009; Flacco et al., 2019).

**FIGURE 1:**
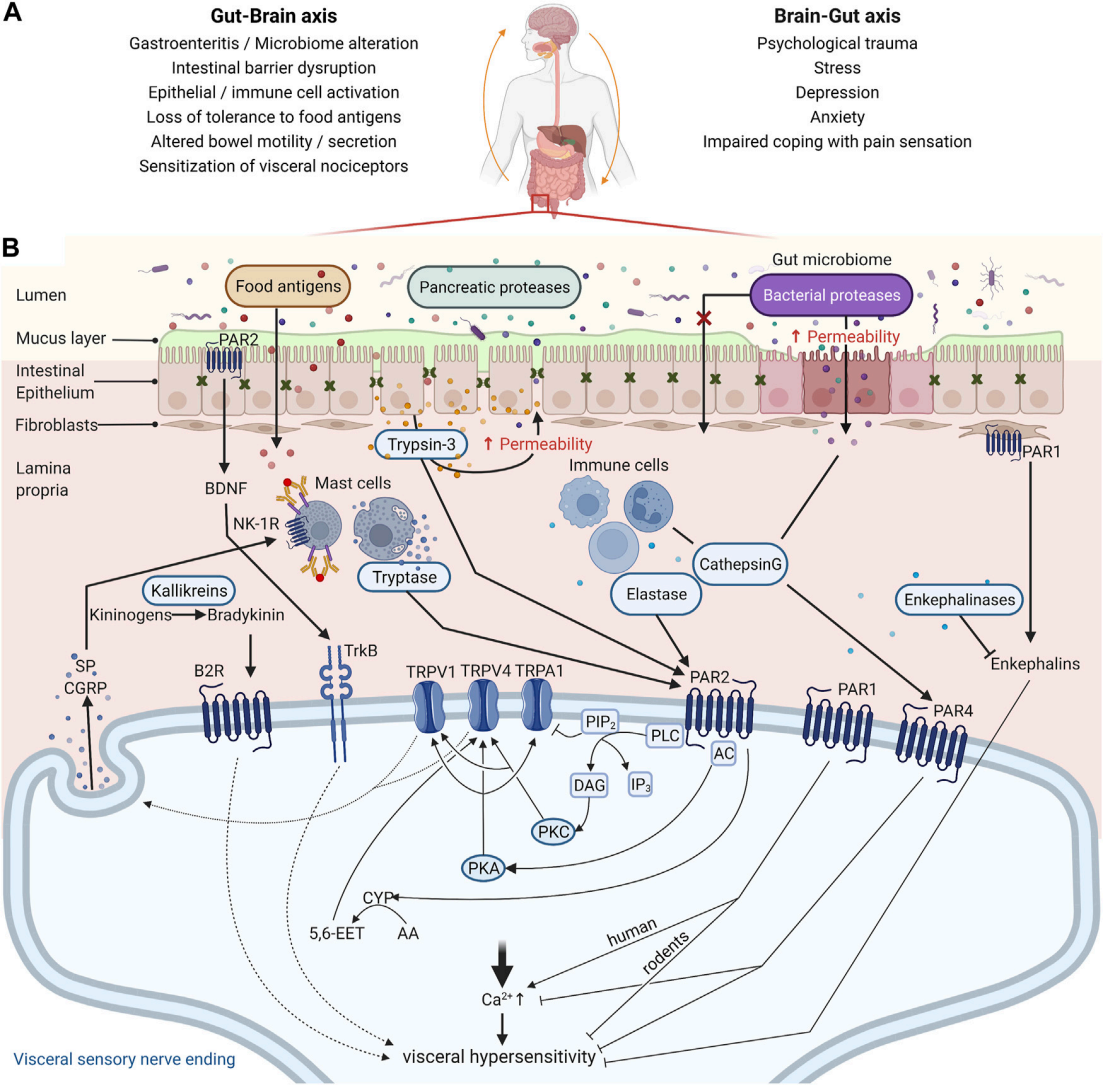
Mechanisms contributing to abdominal pain associated with irritable bowel syndrome (IBS). **(A)** The pathophysiology of IBS is tightly associated with perturbations in the Gut-Brain Axis, which encompasses a bi-directional communication between these organs. Increasing reports indicate that gastroenteritis may promote intestinal barrier disruption, activation of epithelial and immune cells in the gut mucosa, and loss of tolerance to dietary antigens. Upon re-exposure to those dietary antigens, mast cells degranulate and their mediators sensitize nociceptors leading to visceral hypersensitivity. Conversely, IBS is also associated with a higher prevalence of psychiatric disorders such as depression and anxiety, factors that affect coping with pain sensation. A history of psychological trauma and stress are also risk factors for IBS, although the mechanisms involved remain unclear. **(B)** Peripheral sensitization of the gut innervation is a central mechanism underlying abdominal pain in IBS, and proteases are emerging as central mediators in this process. Proteases from the lumen (pancreatic or microbial), epithelial cells (trypsin-3), and immune cells (tryptase, elastase, and cathepsin (G) participate in the disruption of the intestinal barrier and can signal to visceral nociceptors and mediate VHS via protease-activated receptor (PAR) activation. PAR2 activation on the nerve endings of visceral nociceptors induces VHS through TRPV1, TRPV4, and/or TRPA1 sensitization after Gɑs or Gɑq recruitment, depending on the protease. TRPV1 and TRPV4 on sensory neurons also mediate the release of substance P (SP) and Calcitonin gene-related peptide (CGRP), further contributing to the pain signal. Additionally, proteases modulate synthesis and secretion of respectively bradykinin and brain-derived neurotrophic factor (BDNF), compounds found to be involved in inflammatory pain. On the other hand, activation of PAR1 and PAR4 has an analgesic effect in mice, while in humans PAR1 is emerging as pro-nociceptive. For PAR4 this is likely mediated through reducing intracellular calcium levels. Whether PAR1 mediates VHS due to activation of the receptor in the sensory nerve endings or fibroblasts remains to be investigated in animal models of IBS and patient samples. Unlike the other PARs, PAR3 seems to rather act as a cofactor for PAR1 and PAR4 signaling by potentiating the cleavage by proteases like thrombin, but its role still warrants further investigation. Transient Receptor Potential Cation Channel Subfamily V Member 1 (TRPV1) and 4 (TRPV4); Transient Receptor Potential Cation Channel Subfamily A Member 1 (TRPA1); Adenylyl Cyclase (AC); Protein Kinase A (PKA); Phospholipase C (PLC); Protein Kinase C (PKC); Cytochrome P450 Epoxygenase (CYP); Arachidonic Acid (AA); 5,6-epoxy-8Z,11Z, 14Z-eicosatrienoic acid (5,6-EET); Neurokinin-1 Receptor (NK1-R); Bradykinin Receptor B2 (B2R); Neurotrophic Receptor Tyrosine Kinase 2 (TrkB); Phosphatidylinositol 4,5-bisphosphate (PIP2); Inositol 1,4,5-trisphosphate (IP3); Diacyl Glycerol (DAG). Created with BioRender.com.

The symptoms of IBS are likely a manifestation of several contributing mechanisms, as depicted in Figure 1A. Visceral hypersensitivity (VHS), an increased pain perception in the gastrointestinal tract, is a major contributing factor to abdominal pain in IBS (Farzaei et al., 2016). Mechanical, thermal, or chemical information in the gut wall is sensed by extrinsic, primary afferent neurons with cell bodies in the dorsal root ganglia (DRG). The signal is transduced to different somatosensory areas in the brain via secondary neurons in the spinal cord, where it is subject to central processing to be perceived as noxious or not (Vermeulen et al., 2014). Peripheral sensitization of extrinsic, primary afferents is determined by the expression of specific channels that are involved in sensing noxious stimuli (Anand et al., 2007). In particular, the transient receptor potential (TRP) channels, more specifically TRPA1, TRPV1, and TRPV4, were shown to be sensitized in submucosal neurons of rectal biopsies from IBS patients, thereby contributing to VHS (Wouters et al., 2016; Balemans et al., 2017, 2019). Besides histamine, bradykinin, and serotonin, also proteases have emerged as mediators that can sensitize these channels and consequently contribute to VHS in IBS (Farzaei et al., 2016; Brizuela et al., 2021).

Proteases are enzymes that hydrolyze peptide bonds, which can result not only in the inactivation of the target but also in the activation of signaling molecules and receptors. In the gastrointestinal tract, while proteases are best known for their role in food digestion, they are now emerging as pivotal mediators of increased intestinal epithelial permeability and visceral pain (Cenac, 2013; Van Spaendonk et al., 2017). Among the different classes of proteases, serine proteases are most likely to mediate pain since they are predominantly released to the extracellular milieu (Vergnolle, 2016). This family of proteases is characterized by the presence of a nucleophilic serine in the reactive site and is further subdivided into catalytic groups according to substrate preference as chymotrypsin-, trypsin- and elastase-like serine proteases (Ovaere et al., 2009). This review focuses on the role of serine proteases in the pathophysiology of VHS in IBS, as well as the therapeutic potential of their inhibition.

## How Proteases Mediate Visceral Pain in IBS

Proteases can modulate visceral pain through several mechanisms as illustrated in Figure 1B, which are discussed in the following paragraphs. The most extensively studied mechanism to date, is via activation of protease-activated receptors (PARs). PARs belong to a family of cell-surface signaling proteins called G protein-coupled receptors and the four members of the PAR family, PAR1-4, are expressed throughout the body, including various cell types in the intestines, such as epithelial cells, mast cells, and neurons (Vergnolle, 2005). Different proteases can cleave the receptors at different sites and thereby induce different signaling pathways or even inactivate the receptor (Gottesman, 2021). Trypsin and tryptase activate canonical signaling after cleavage of PAR2, leading to G-protein mediated signaling via downstream messengers such as PKC (Dai et al., 2004; Amadesi et al., 2006; Grant et al., 2007). Cathepsin S and elastase cleave elsewhere and activate biased signaling, which includes G-protein mediated activation of cAMP and PKA (Zhao et al., 2015). Hence, the profile of active proteases within a tissue microenvironment is a major determinant of the downstream PAR signaling taking place, with direct consequences for nociception.

Evidence from pre-clinical studies has indicated that proteases can promote VHS in mice via PAR2-mediated sensitization of TRPA1 and TRPV4. Intracolonic administration of PAR2 activating peptide causes visceral hyperalgesia in wild-type mice, but not in TRPA1 knock-out mice, suggesting that TRPA1 is needed to mediate PAR2-induced hyperalgesia (Cattaruzza et al., 2010). Additionally, using TRPV4-targeted siRNA and PAR2 knock-out mice, Cenac and others show that colonic biopsy supernatants from IBS-D patients induce VHS when administered in the colon, in a TRPV4-dependant mechanism by activating PAR2 in sensory neurons (Cenac et al., 2015). A link between PAR2 activation and TRPV1 sensitization has also been shown in murine DRG neurons and models of somatic pain, but this remains to be explored further in visceral pain (Amadesi et al., 2006). PAR1 activation induces antinociceptive effects and even appears to prevent visceral hyperalgesia in mice (Asfaha et al., 2002; Kawao et al., 2004; Martin et al., 2009). However, studies on human enteric and DRG neurons showed that colonic biopsy supernatant from IBS patients could activate the neurons via PAR1, but not PAR2 (Mueller et al., 2011; Kugler et al., 2012; Buhner et al., 2018; Desormeaux et al., 2018). This might suggest very distinct roles for PAR1 and PAR2 in humans versus rodents and might be explained in part by the fact that the class of serine proteases has many lineage-specific differences between humans and mice (Puente et al., 2003). PARs also have other ways to signal than calcium mobilization that would be of interest to investigate in regard to TRP sensitization (Zhao et al.,2014a; 2014b; Sostegni et al., 2015). Interestingly, expression of both PAR1 and PAR4, also suggested to be antinociceptive, was lowered in IBS patients compared to healthy controls, while PAR2 levels were unchanged (Bian et al., 2009; Zhao et al., 2012). To date, there is no data showing co-expression of TRP channels and PARs on human DRGs or sensory nerve endings in human colonic biopsies. Nevertheless, both TRP channels and PARs are broadly expressed in nociceptive neurons; therefore, their co-expression on nerve endings innervating the gut is quite certain (Cenac, 2013; Balemans et al., 2017; Desormeaux et al., 2018).

Proteases can also convert molecules that are active on pain pathways. Bradykinin for example, a potent proinflammatory mediator, is generated through cleavage of kininogens by kallikreins, which are serine proteases, and can induce hyperalgesia via TRPV1 and TRPA1 sensitization (Mizumura et al., 2009). The contribution of the kallikrein-kinin pathway in experimental acute colitis is without controversy, however, there is a lack of studies investigating the role of this mechanism in VHS (Stadnicki, 2011). Endogenous opioids are modulators of nociceptive signaling promoting analgesia of which the activity is also regulated by serine proteases. Enkephalins, one of the endogenous opioid families, are known to promote ion and water absorption in the gut, as well as play a role in visceral nociception (Mosinska et al., 2016). Aminopeptidase N (ANPEP), a metallopeptidase, is an important enzyme involved in the degradation of enkephalins. In colonic biopsies taken from inflamed regions of patients with inflammatory bowel disease (IBD), increased levels of enkephalins were described but protease activity levels were not assessed (Owczarek et al., 2011). To date, reports regarding levels of ANPEP or endogenous opioids in IBS patients are lacking, but levels of opioid receptor expression were significantly lower in the colon of diarrhea-predominant IBS patients compared to healthy controls (Zielińska et al., 2015).

## Proteases in Visceral Hypersensitivity

An increase in serine protease activity, mainly trypsin-like, in colonic tissue or tissue supernatants of IBS patients compared to healthy controls has been reported by several groups (Barbara et al., 2004; Cenac et al., 2007; Buhner et al., 2009; Rolland-Fourcade et al., 2017; Aguilera-Lizarraga et al., 2021). Accordingly, elevated tryptase and trypsin mRNA and protein levels were shown (Zhao et al., 2012; Liang et al., 2016). The increase in tryptase levels was correlated with a higher number of mast cells in the mucosa by some groups (Barbara et al., 2004; Bian et al., 2009; Buhner et al., 2009) although others reported no increase in mast cell count (Cenac et al., 2007; Wouters et al., 2016). Most recently, we provided compelling evidence that mast cells in the rectal mucosa of IBS patients are more sensitized with IgE, thereby more prone to degranulation and tryptase release upon antigen stimulation (Aguilera-Lizarraga et al., 2021). Trypsin-3, as revealed by *in situ* zymography, is expressed by the intestinal epithelium and upregulated in IBS (Rolland-Fourcade et al., 2017). Thrombin, recently identified as upregulated in IBD patients (Denadai-Souza et al., 2018), did not differ, at least in expression levels, between IBS patients and controls (Bian et al., 2009). Interestingly, proteome analysis with LC-MS/MS (liquid chromatography with tandem mass spectrometry) revealed upregulation of elastase-like and not trypsin-like serine proteases in colonic biopsy supernatants from IBS patients (Buhner et al., 2018). However, no complementary experiments were done to confirm this finding. Also, in feces of IBS patients, researchers found an increase in serine protease activity from either the patient or the intestinal bacteria (Annaházi et al., 2009; Tooth et al., 2014; Edogawa et al., 2020). Although these enzymes are less likely to play a direct role in the sensitization of intestinal nociceptors since they would first have to cross the epithelial barrier, they might be involved in barrier dysfunction (Steck et al., 2012; Lomax et al., 2019).

## Protease Inhibitors in Visceral Hypersensitivity

With increasing evidence that serine proteases contribute to the pathophysiology of IBS, also the search for effective protease inhibitors as therapeutic agents has started. Targeting proteases has resulted in several great success stories in other conditions such as HIV and cardiovascular diseases but it remains a challenging endeavor overall (Turk, 2006; Drag, 2010). To date, no protease inhibitors are marketed or in clinical trials for IBS and only few studies have explored the effects of serine protease inhibitors in experimental models of VHS.

Nafamostat mesylate, also called FUT-175, is a short-acting, synthetic inhibitor of serine proteases with poor oral bioavailability that has been approved in Japan as a treatment for acute pancreatitis and disseminated intravascular coagulation (Cao et al., 2008). FUT-175 could prevent development of VHS in WT mice when co-administered intracolonic with IBS supernatants (Cenac et al., 2007). Additionally, a single intraperitoneal injection of FUT-175 normalized visceral sensitivity in post-colitis rats (Ceuleers et al., 2018). Camostat mesylate, or FOY-305, has similar properties and indications as FUT-175. Intragastric pretreatment with FOY-305 could prevent acute stress-induced VHS in rats (Zhao et al., 2011). Both inhibitors target a broad-spectrum of serine proteases, have anticoagulation activity and are associated with rare, but important side effects such as hyperkalemia, as well as mild complications like headache (Sawada et al., 2016; Quinn et al., 2022). It is important to emphasize that the broad range of targets and intravenous administration (for FUT-175) are crucial determinants of the risk for side effects. Improved targeting of the inhibitors is definitely needed when considering these compounds as treatment options for a chronic and not life-threatening condition such as IBS. Alternatively, oral compounds that act locally and are not systemically absorbed are most likely the safest approach with minimal risk of side effects.

Two newly developed serine protease inhibitors, UAMC-00050 and UAMC-01162, have a more favorable inhibitory profile with a limited inhibition of proteases related to the blood coagulation cascade, but a good inhibition of tryptase (Joossens et al., 2007). Neither had an effect on visceral sensitivity in healthy control animals but successfully reversed VHS in post-colitis rats in the highest dose used (1 mg/kg UAM-00050; 2.5 mg/kg UAMC-01162) (Ceuleers et al., 2018). Plant-derived Bowman-Birk inhibitors are specific toward trypsin and chymotrypsin and are present in vegetables like soybeans. Oral administration of soy germ extract for 15 days resulted in a decrease in visceral sensitivity, fecal protease activity, and PAR2 expression in a stress-induced rat model for VHS (Moussa et al., 2013). However, soy germ extract also contains phytoestrogens and an estrogen-receptor antagonist reversed all effects, except the decrease in fecal proteolytic activity (Moussa et al., 2013). The positive effect of soy germ extract is therefore likely a result of the combination of estrogen-receptor and PAR2 mediated pathways. A randomized, double-blind, placebo-controlled trial done in patients with ulcerative colitis indicated that soy extract is safe although not very effective (Lichtenstein et al., 2008).

Another therapeutic strategy is to increase the bioactive life-span of endogenous opioids in the gut and thereby relieve abdominal pain. Agonists of the µ-opioid receptor are already on the market for diarrhea-predominant IBS, e.g. loperamide, but these drugs can cause severe secondary constipation and do not adequately resolve abdominal pain (Moayyedi et al., 2019). Inhibiting opioid degrading enzymes would have an effect that is restricted in time and space, allowing better control of pharmacological activity. Additionally, repeated administration of RB101, the first dual enkephalinase inhibitor, did not induce tolerance or physical dependence in early animal studies, unlike opioid receptor agonists such as morphine (Noble et al.,1992a; 1992b). Sialorphin, a natural enkephalinase inhibitor, was recently studied for its analgesic potential in a mouse model of visceral pain induced by colorectal distension (Fabisiak et al., 2018). They showed that subcutaneous administration of sialorphin increased the pain threshold comparable to the opioid Leu-enkephalin. Nevertheless, further and more targeted research investigating the potency and side effects of these inhibitors is necessary.

Protease inhibitors with greater specificity have not been tested for their effects on VHS, mainly because the exact targets still need to be identified. However, the following inhibitors might be of interest to explore further. Leupeptin, an organic compound produced by many Actinomycetales bacteria in the human intestinal tract, targets serine proteases (trypsin, plasmin, and kallikrein) and cysteine proteases (papain and cathepsin B), but not thrombin, elastase, or chymotrypsin. This inhibitor was used by Rolland-Fourcade et al. to demonstrate that lipopolysaccharide-stimulation of intestinal epithelial cells results in basolateral release of trypsin-like proteases (Rolland-Fourcade et al., 2017). APC-366 is a tryptase-specific inhibitor and was employed recently to discriminate tryptase activity from total trypsin-like activity (Aguilera-Lizarraga et al., 2021). Initially, it was developed to treat asthma, allergy, and colitis, but it lacked potency and selectivity. This inhibitor also has high affinity for thrombin, a pivotal enzyme in blood coagulation, thus when not applied locally, APC-366 could lead to bleeding. APC-2059 had improved specificity and seemed to be useful as adjuvant therapy in ulcerative colitis in an open-label, phase two clinical study (Tremaine et al., 2002). However, it needed to be injected and was not developed further.

This indicates that drugability is a critical aspect that requires a lot of attention. For compound UAMC-00050 the efficacy decreased when given locally via an enema versus intraperitoneal injection (Ceuleers et al., 2018; Hanning et al., 2021). Plasma concentrations were very low after intracolonic administration and on average only 5.4% of the plasma concentration after intravenous administration although a five times larger dose was used for local application. This suggests that the inhibitor mainly stays in the lumen or intestinal mucosa. The animals were observed for 3 h after administration of the drug and no side effects were noted. Nonetheless, it would be more relevant for clinical trials if the compounds can be taken orally. Giardina et al. developed a novel inhibitor of human b-tryptase with good oral bioavailability (Giardina et al., 2018). As a dimeric inhibitor, it bridges two active sites resulting in increased affinity and specificity compared to monomeric controls. But unlike previously reported polyvalent inhibitors, this one contains a disiloxane linker, which greatly improves the bioavailability after oral administration. This compound would be of great interest to explore in the context of IBS. The same applies for the use of recombinant lactic acid bacteria to deliver serine protease inhibitors to the intestinal mucosa (Bermúdez-Humarán et al., 2015). However, only biologicals can be delivered via this system and these tend to have a broader selectivity.

## Towards More Selective Protease Targeting

The two major factors hampering the development of specific protease inhibitors are the superficial identification of overactive serine proteases in this disorder, as well as the limited mapping of substrate preferences of relevant proteases. Activity-based probes (ABPs) provide a solution for the first obstacle. The reactive group of the probe binds specifically and irreversibly to the proteases of interest. This allows identification of the bound proteases by mass spectrometry after affinity-based purification. The use of these probes is slowly increasing in the field of inflammatory and functional bowel diseases, which can only be encouraged further (Mayers et al., 2017; Denadai-Souza et al., 2018; Anderson et al., 2019; Solà-Tapias et al., 2020). To tackle the second obstacle; the hybrid combinatorial substrate library (HyCoSuL) using natural and unnatural amino acids, is an efficient way to find highly selective protease substrates (Kasperkiewicz et al., 2014). A HyCoSuL for serine proteases would consist of three sublibraries of tetrapeptides with a fixed amino acid at P1 and amino acid mixtures at the P4-P2 positions as well as an ACC (7-aminocoumarin-4-acetic acid) fluorescent tag occupying the P1’ position. Once the tetrapeptide is cleaved, the ACC produces a fluorescent signal.

HyCoSuL has proven to be a valuable tool to study protease substrate preference, however, it is limited to the amino acid residues N-terminal to the cleavage site (Poreba et al., 2018; Anderson et al., 2019). Internally quenched fluorescent substrates are an excellent way to study the specificity of both the prime and non-prime pockets but are inefficient for large screenings. A very good tool for screening a large (up to 10^10^) and diverse pool of substrates is phage display technology (Kehoe, 2005; Caberoy et al., 2011). Random peptides are expressed on the phage surface and subjected to protease cleavage followed by phage amplification if successfully cleaved. Thus, optimal substrates are enriched over multiple rounds of selection. A major drawback is that the substrates are label-free, so they need to be resynthesized with an appropriate reporter group to compare the kinetics of the best substrates. Label-free substrates are not per se a bad thing; not having a label right next to the cleavage site means it also cannot influence the enzyme-substrate binding interactions. Other approaches to study substrate specificity without the use of reporter tags utilize mass spectrometry (O’Donoghue et al., 2012; Wood et al., 2017; Lapek et al., 2019). A beautiful example by De Bruyn et al. in an acute- and a post-colitis rat model illustrates this is a valuable way to investigate the identity and cleavage patterns of proteases in biological samples (De Bruyn et al., 2021). By applying this method, the authors demonstrated that tryptase and trypsin-3 also unmask PAR1. Nevertheless, also this technique is somewhat biased and requires prior knowledge of the proteases that are being studied. It is important to note that these methods do not exclude each other, on the contrary, they are complementary and provide extra strength where there is overlap.

**FIGURE 2:**
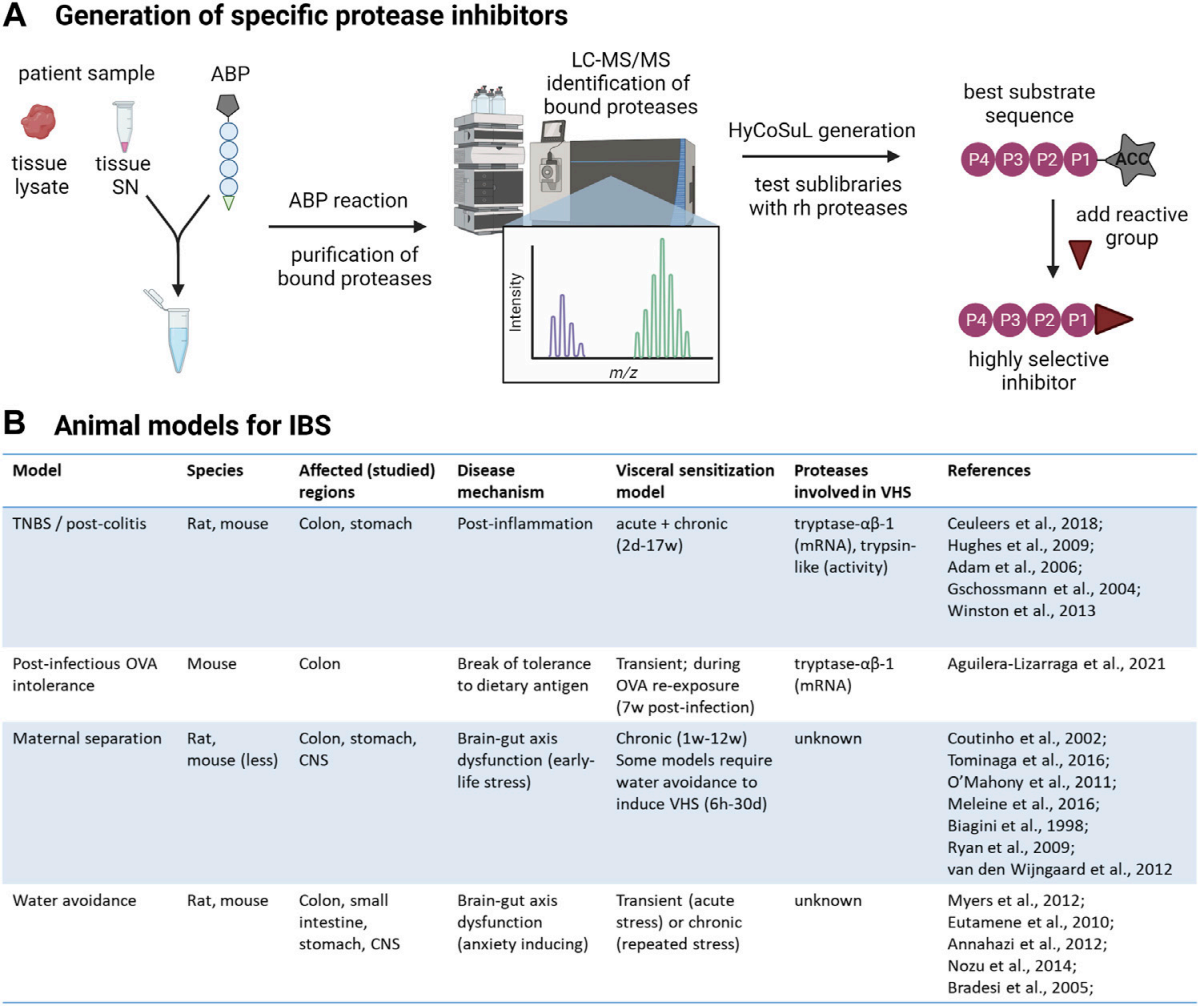
From protease identification to inhibitor design. **(A)** Workflow to develop highly selective protease inhibitors. First, serine proteases present in patient samples, whole tissue lysate or supernatant (SN) containing secreted mediators, will be labeled with desthiobiotin-tagged activity-based probes (ABPs) specific for this class. The desthiobiotin-tag of the ABP is represented as a pentagon, the circles represent the recognition element, and the reactive group that will bind the attacking protease is represented as a triangle. The bound proteases will be isolated with paramagnetic beads conjugated to streptavidin and analyzed by LC-MS/MS. This will unambiguously inform the researcher about the identity of all active serine proteases in the sample and which ones are increased in IBS patients compared to controls. Next, hybrid combinatorial substrate libraries (HyCoSuL) will be developed to determine the substrate specificities of the identified protease(s). For serine proteases, three tetrapeptide sublibraries are made with a fixed amino acid at the P1 position, according to the serine protease of interest. The protease will attack the bond between the amino acid at P1 and the fluorogenic reporter (7-aminocoumarin-4-acetic acid, ACC) at the P1′ position so that hydrolysis can be measured in a plate reader. In each sublibrary, one of the three remaining positions in the tetrapeptide (P2-P4) has a fixed amino acid while the remaining positions contain an equimolar mixture of amino acids. Ultimately, the optimal substrates can be determined using recombinant human (rh) proteases by combining the results from all sublibraries. With prior knowledge about the specificity of the protease, the palette of amino acids can be customized to minimize the number of sublibraries. The best substrate sequence will be used as a scaffold to synthesize highly specific protease inhibitors. **(B)** Animal models of IBS to test the effectiveness of serine protease inhibitors on abdominal pain. TNBS: Trinitrobenzene sulfonic acid; OVA: ovalbumin; CNS: central nervous system (Adam et al., 2006; Annaházi et al., 2012; Biagini et al., 1998; Bradesi et al., 2005; Coutinho et al., 2002; Eutamene et al., 2010; Gschossmann et al., 2004; Hughes et al., 2009; Meleine et al., 2016; Myers and Greenwood-Van Meerveld, 2012; Nozu et al., 2014; O' Mahony et al., 2011; Ryan et al., 2009; Tominaga et al., 2016; van den Wijngaard et al., 2012; Winston and Sarna, 2013). Created with BioRender.com.

Combining target identification via ABPs and inhibitor design via HyCoSuL should provide a reliable and accessible toolbox to design highly specific protease inhibitors for the management of abdominal pain in IBS (Figure 2A). Testing the effectiveness and safety of promising serine protease inhibitors should be done in animal models of IBS to maximize translational value (Figure 2B). Although these models inherently are limited in reflecting the complex disease mechanisms underlying IBS, they are able to induce VHS, an important factor in IBS and the target of these protease inhibitors (Accarie, 2020). It must be noted that the research investigating the role of serine proteases in VHS in these models is in its infancy and the translational value of the results requires further exploration.

## Conclusion

The investigation of protease dysregulation in gastrointestinal diseases, and more specifically in VHS and IBS, has definitely increased in recent years, bringing protease inhibition to the forefront of one of the most promising novel therapeutic strategies to explore. However, three important factors need to be addressed to advance serine protease inhibitors as a treatment for VHS in IBS. The first is the limited knowledge of the targets. The dysregulated proteases need to be characterized unambiguously and on a patient level, an endeavor that can be achieved with activity-based probes. Secondly, as underlined in this review, proteases have many important physiological roles and any inhibition will have to be carefully targeted to limit side effects. Accurate mapping of substrate specificities of the proteases of interest with a technique like HyCoSuL will facilitate and accelerate the development of highly specific serine protease inhibitors. The third and final limiting factor is that the current oral bioavailability of serine protease inhibitors is poor and improvement of formulations favoring local delivery should be prioritized to give the inhibitors a higher chance to succeed in clinical trials for IBS.
